# From organ to cell: Multi-level telomere length assessment in patients with idiopathic pulmonary fibrosis

**DOI:** 10.1371/journal.pone.0226785

**Published:** 2020-01-07

**Authors:** Aernoud A. van Batenburg, Karin M. Kazemier, Matthijs F. M. van Oosterhout, Joanne J. van der Vis, Hendrik W. van Es, Jan C. Grutters, Roel Goldschmeding, Coline H. M. van Moorsel

**Affiliations:** 1 Department of Pulmonology, St Antonius ILD Center of Excellence, St Antonius Hospital, Nieuwegein, the Netherlands; 2 Laboratory of Translational Immunology, University Medical Center Utrecht, Utrecht, Netherlands; 3 Department of Respiratory Medicine, University Medical Center Utrecht, Utrecht, the Netherlands; 4 Pathology–DNA, Department of Pathology, St Antonius ILD Center of Excellence St Antonius Hospital, Nieuwegein, The Netherlands; 5 Department of Clinical Chemistry, St Antonius ILD Center of Excellence, St Antonius Hospital, Nieuwegein, the Netherlands; 6 Department of Radiology, St Antonius ILD Center of Excellence, St Antonius Hospital, Nieuwegein, the Netherlands; 7 Division of Heart and Lungs, University Medical Center Utrecht, Utrecht, the Netherlands; 8 Department of Pathology, University Medical Center Utrecht, Utrecht, the Netherlands; University of Newcastle, UNITED KINGDOM

## Abstract

**Rationale:**

A subset of patients with idiopathic pulmonary fibrosis (IPF) contains short leukocyte telomeres or telomere related mutations. We previously showed that alveolar type 2 cells have short telomeres in fibrotic lesions. Our objectives were to better understand how telomere shortening associates with fibrosis in IPF lung and identify a subset of patients with telomere-related disease.

**Methods:**

Average telomere length was determined in multiple organs, basal and apical lung, and diagnostic and end-stage fibrotic lung biopsies. Alveolar type 2 cells telomere length was determined in different areas of IPF lungs.

**Results:**

In IPF but not in controls, telomere length in lung was shorter than in other organs, providing rationale to focus on telomere length in lung. Telomere length did not correlate with age and no difference in telomere length was found between diagnostic and explant lung or between basal and apical lung, irrespective of the presence of a radiological apicobasal gradient or fibrosis. Fifteen out of 28 IPF patients had average lung telomere length in the range of patients with a telomerase (*TERT*) mutation, and formed the IPF_short_ group. Only in this IPF_short_ and TERT group telomeres of alveolar type 2 cells were extremely short in fibrotic areas. Additionally, whole exome sequencing of IPF patients revealed two genetic variations in *RTEL1* and one in *PARN* in the IPF_short_ group.

**Conclusions:**

Average lung tissue telomere shortening does not associated with fibrotic patterns in IPF, however, approximately half of IPF patients show excessive lung telomere shortening that is associated with pulmonary fibrosis driven by telomere attrition.

## Introduction

Idiopathic pulmonary fibrosis (IPF) is a life-threatening disease of the lung, caused by progressive decay of alveolar epithelium and parenchymal scarring. Median survival of IPF patients after diagnosis is approximately 4 years [1,2].

IPF is a heterogeneous disease that can occur sporadically or in the context of familial disease. A major genetic cause underlying familial disease comprise of mutations in telomerase related genes involved in the maintenance of telomere length [3–6]. Mutations are most commonly found in the gene encoding telomerase reverse transcriptase (*TERT*). Defects in telomerase result in extremely short telomeres and lead to abnormal DNA repair, or DNA degradation, and eventually to cellular senescence, a phenomenon known to be involved in acute wound healing as well as in lung fibrosis [7–12]. In families carrying *TERT* mutations, pulmonary fibrosis was shown to be the most common disease manifestation [13]. Moreover, it was shown that not the presence of the mutation itself, but the resulting short telomeres were associated with development of fibrosis [14]. IPF cases and patients with telomere related gene mutations have short leukocyte telomere length, but present with high inter-individual variation. It is therefore thought that in a subgroup of patients with IPF telomere maintenance is a key to disease pathogenesis whereas in others it is not [15–18].

During healthy ageing, leukocyte telomere length declines approximately 20–30 base pairs per year due to shortening per cell division [19]. In other organs, cell turnover rates are different and therefore telomere length may vary between organs [20]. Several studies discovered differences in telomere length between human organs [20–23]. However, the lung was never included. One primate study showed that in healthy macaques telomeres were excessively shorter in a subset of organs, including the lungs [24].

Not only variation in telomere length between organs may exist, also variation within organs may be present. IPF lungs are typically characterized by a usual interstitial pneumonia (UIP) pattern on histology and high-resolution computed tomography (HRCT). The histological pattern of UIP consists of healthy air-containing non-fibrotic areas juxtaposed to dense fibrotic areas with fibroblast foci and honeycombing features. Previously we showed that telomere shortening in alveolar type 2 cells associates with fibrotic lesions [25]. On HRCT, IPF lungs typically show an apicobasal gradient, in which fibrosis is most abundant subpleurally in the basal lung fields [1]. In control lungs, in which such a gradient is absent, it was recently found that telomere length in the basal fields was significantly shorter than in the apical fields of the same lung [26]. It is unknown whether telomere shortening in the IPF lung associates with the apicobasal gradient.

In this study, we showed that average telomere length does not associate with lung localization in apical and basal regions or disease duration. Furthermore, measurements of telomere length enabled us to identify a subgroup of sporadic IPF patients with lung telomere length similar to that found in patients carrying a telomerase mutation.

## Materials and methods

### Patient and tissue selection

In total 82 subjects were included in the study, consisting of 49 patients with sporadic IPF, 18 pulmonary fibrosis patients with a *TERT* mutation, and 15 controls. The study was approved by Medical research Ethics Committees United (MEC-U) of the St Antonius Hospital (approval number W14.056 and R05-08A). All patients were recruited when visiting the St Antonius Hospital for ILD care and provided written informed consent. Research was conducted at the department of pulmonology of the St Antonius Hospital Nieuwegein and at the department of pathology of the University Medical Center Utrecht. Diagnoses were made according to ATS/ERS/JRS/ALAT guidelines [1,2] by a multidisciplinary team including an experienced pulmonologist (JCG), radiologist (HWvE) and pathologist (MFMvO) at St. Antonius ILD centre of excellence Nieuwegein, The Netherlands.

Biopsy specimens originated from three different sources: group 1 consisted of autopsy specimens, group 2 of diagnostic biopsies, and group 3 of material from explant lung (S1 Fig). Formalin-fixed paraffin-embedded (FFPE) lung slides were cut from residual biopsies. Surgical biopsy specimens from IPF and TERT subjects, obtained between 1994 and 2015, were randomly included if histological usual interstitial pneumonia (UIP) characteristics were present. Biopsies were taken subpleurally and showed a patchy fibrotic/non-fibrotic pattern, fibroblast foci and honeycombing. Furthermore, in the IPF group, familial subjects were excluded and IPF patients were screened negative for mutations in TERT, TERC, surfactant protein C (SFTPC), surfactant protein A2 (SFTPA2) exon 6 and TRF1-Interacting Nuclear Factor 2 (TINF2) exon 6. Relevant demographic details of patients with a diagnostic biopsy are presented in Table 1. Additional information on patient selection and clinical data collection are provided in an online data supplement (S1 File and S1 Table).

**Table 1:** Characteristics of patients with a diagnostic biopsy.

	*IPF*_*normal*_	*IPF*_*short*_	*TERT*
*N*	17	15	17
*Male/Female*	16/1	13/2	14/3
*Mean Age at diagnosis (SD)*	61.2 (9.1)	58.8 (10)	58.7 (9.8)
*Mean FVC%pred (SD)*	67.9 (23.7)	73 (19.8)	81 (15.6)
*Mean DLCO%pred (SD)*	47.9 (20.3)	44.9 (11.1)	43.6 (6.2)
*Smoking status (CS*:*FS*:*NS*:*U)*	0:11:5:1	2:10:1:2	0:14:3:0
*Pack years (SD)*	16.4 (15.6)	23.9 (22.5)	18 (15.5)

IPF = Idiopathic Pulmonary Fibrosis; FVC = Forced Vital Capacity; DLCO = Diffusing Capacity of the Lungs for Carbon Monoxide. Pred = Predicted; IPF_short_ = lung telomere length in the same range as TERT patients: < 0.857; IPF_normal_ = lung telomere length above the range of TERT patients: > 0.857; CS = Current Smoker; FS = Former Smoker; NS = Never Smoker; U = Unknown.

No significant differences were present between patient groups (Kruskal-Wallis multiple comparison tests).

### Tissue preparation and fluorescence in situ hybridization

FFPE biopsies were prepared, stained and analysed for telomere signals in alveolar type 2 (AT2) cells, using fluorescence *in situ* hybridization (FISH) as described previously [27]. Immunofluorescent staining of proSP-C, a protein exclusively produced by AT2 cells, was used for identification of AT2 cells. A summary is provided in an online data supplement (S1 File).

### Telomere length measurements by MMqPCR in FFPE tissue and peripheral blood leukocytes

DNA was isolated from FFPE samples and T/S ratios were measured as described previously [28]. The T/S ratio is a measure for average telomere length in biopsy or peripheral blood leukocytes obtained by MMqPCR and is proven to be a sensitive method to discriminate between patients with high and low telomere length signals [15,29]. The overall mean coefficient of variation was 2.5 and only samples with a coefficient of variation below 10% were included. A summary of the MMqPCR protocol is provided in an online data supplement (S1 File).

### Whole exome sequencing

DNA extracted from peripheral blood leukocytes of all subjects with sporadic IPF was obtained for whole exome sequencing (WES) at Novogene (Hong Kong, China) using the Agilent SureSelect Human All Exon V6 kit (Agilent Technologies, Santa Clara, CA, USA) on an Illumina PE150 sequencing platform (Illumina, San Diego, California, USA) according to standard protocol. Additional detail on analysis of the sequence data is provided in an online data supplement (S1 File).

### Statistical analysis

Statistical significances were computed using non-parametric tests in GraphPad Prism version 7 (GraphPad Software, San Diego, CA, USA). Telomere length differences were determined by Mann-Whitney tests and a combined Kruskall-Wallis and Dunn’s multiple comparisons tests. Paired data were computed using a Wilcoxon matched-pairs signed rank analysis. Spearman’s rank coefficient was used to calculate correlations.

## Results

### In three IPF patients, organ telomere length is shortest in lung

**Fig 1:**
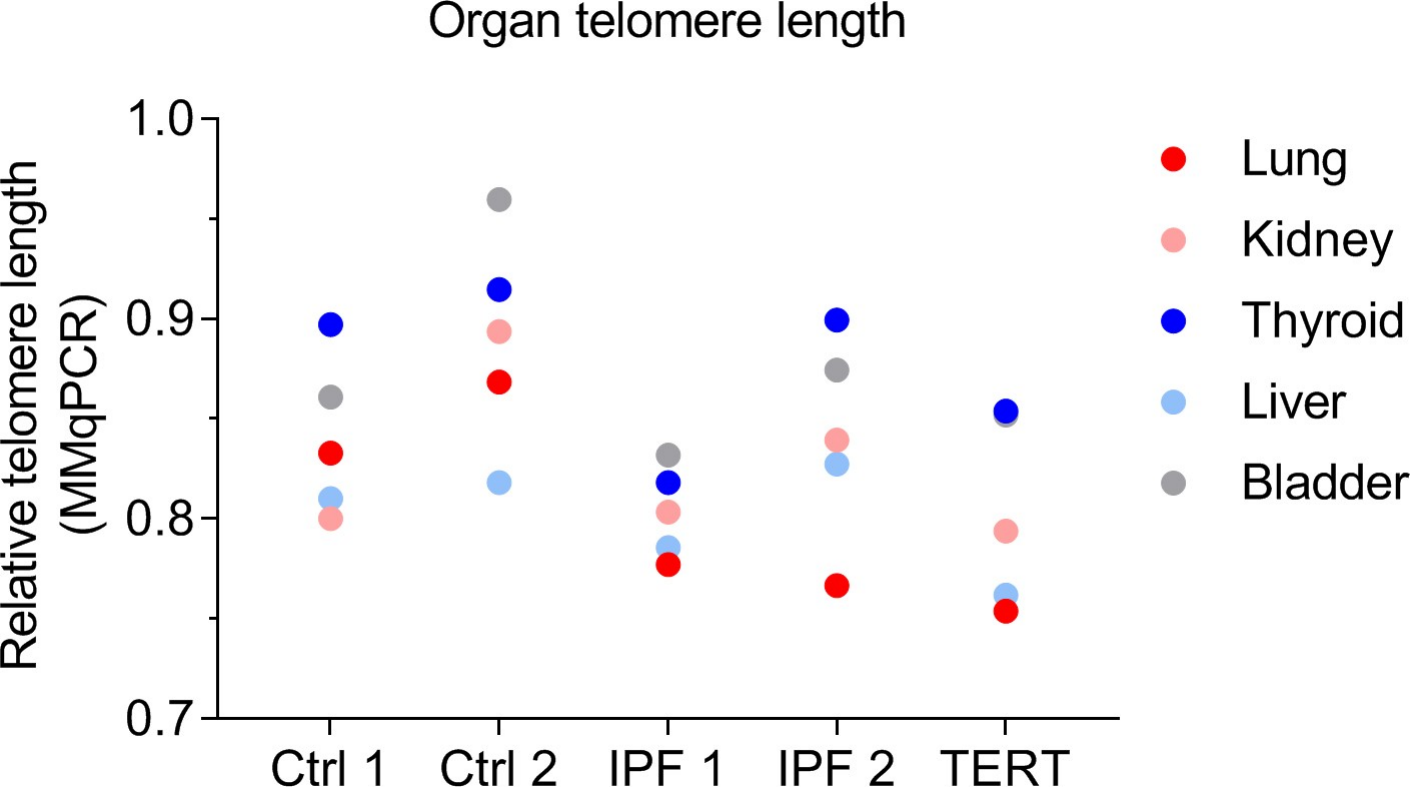
Organ telomere length per subject. Biopsy telomere length measurements by MMqPCR in lung, kidney, thyroid, liver and bladder tissue in 2 controls (Ctrls), 2 spradic IPF patients and one pulmonary fibrosis case with a *TERT* mutation. In patients with pulmonary fibrosis the shortest telomere length was found in lung tissue.

To compare telomere length between fibrotic lung and other organs, we measured average telomere length in lung, kidney, thyroid, liver and bladder of two age and sex matched controls, two sporadic IPF patients and one IPF patient carrying a *TERT* mutation using MMqPCR. In the three patients with pulmonary fibrosis shortest telomere length was present in lung tissue (Fig 1).

### IPF lung telomere length does not correlate with age

**Fig 2:**
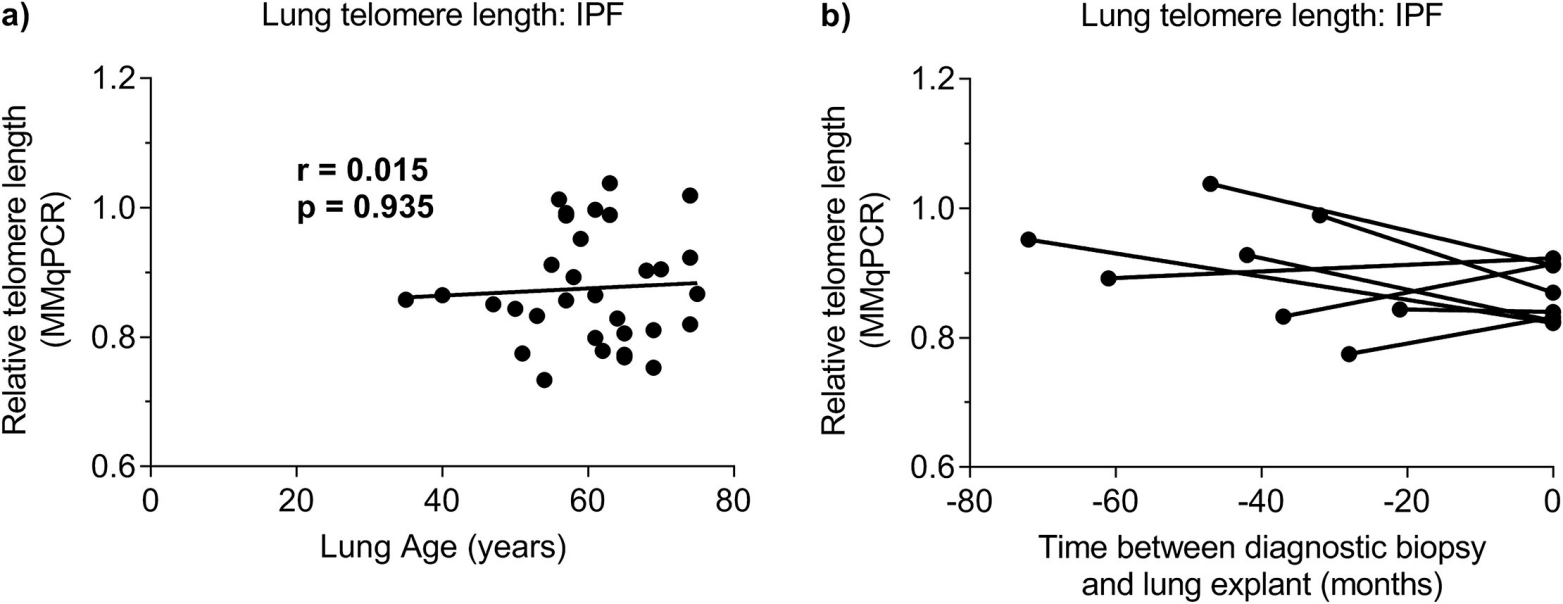
Lung telomere length in IPF. (**a**) Telomere length measurements in lungs of 32 subjects with IPF by MMqPCR. No correlation was found (Spearman correlation; r = 0.015, p = 0.935). (**b**) Comparison of telomere length between 8 diagnostic biopsy and 8 explant lung specimens. Samples belonging to the same patient are connected. Wilcoxon matched-pairs signed rank tests showed no differences in telomere length between samples (p = 0.25).

Lung telomere length of diagnostic biopsy specimens from sporadic IPF patients was measured in a cohort with an age ranging between 35 and 75 years old (n = 32; median age = 61 years old) using MMqPCR. In sporadic IPF no correlation was present between lung telomere length and age (r = 0.015, p = 0.935; Fig 2A). In control lung, correlation analysis showed a trend towards significance between increasing age and shortening of lung telomere length (n = 18, r = -0.4, p = 0.115; S2 Fig).

### Average lung telomere length remains the same during disease

To assess if telomere length changes during IPF disease evolution, we compared MMqPCR lung telomere length of eight diagnostic biopsy specimens with that of explant lung specimens with end-stage fibrosis, taken from the same lobe of the same patients. A median of 45 months passed between the diagnostic biopsy and the transplantation procedure. No difference was found between diagnostic biopsy and explant lung telomere length (p = 0.251; Fig 2B).

### Telomere shortening is not associated with the apicobasal UIP gradient

**Fig 3:**
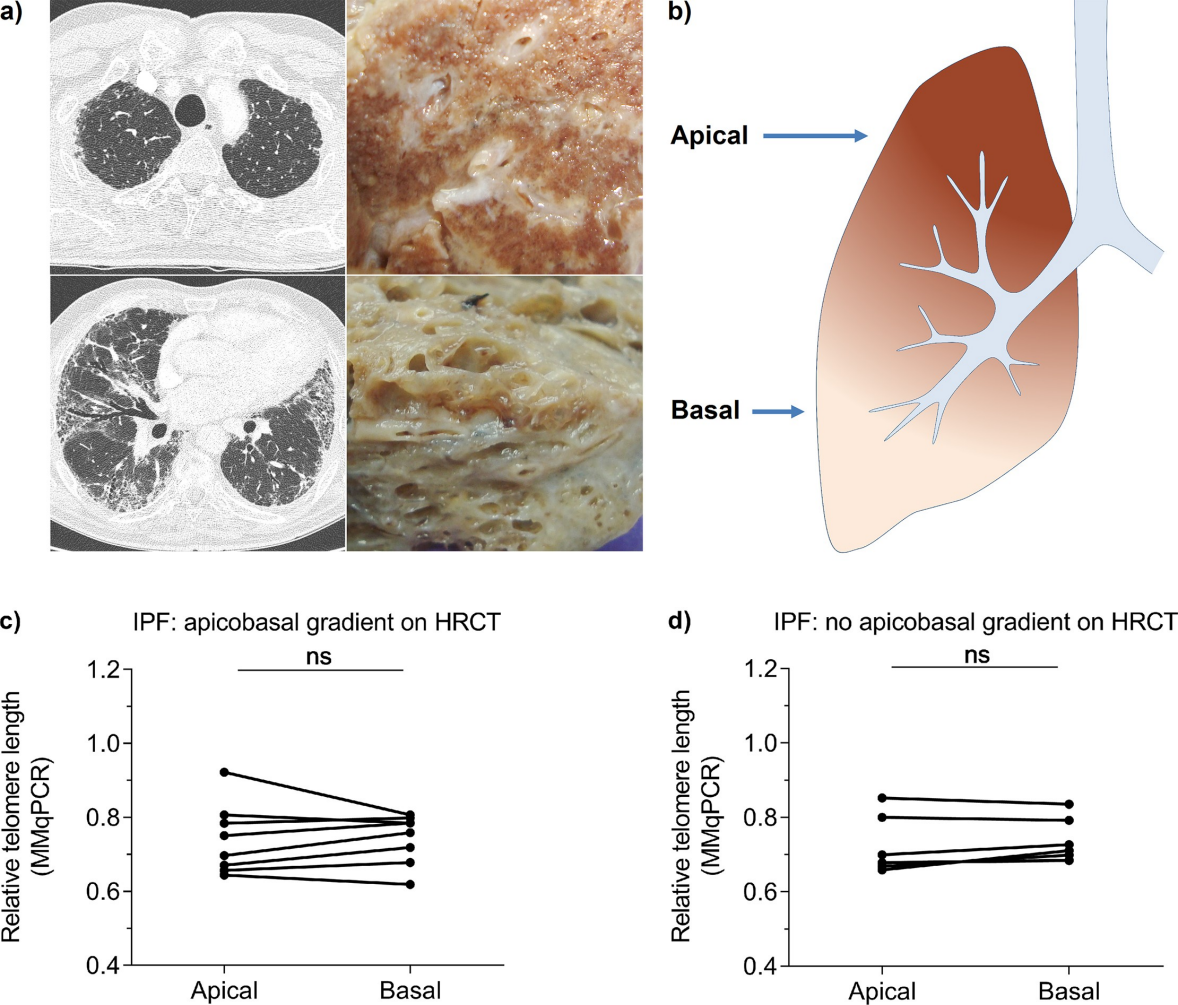
Telomere shortening is not associated with apical or basal localisation in whole explant lung. (**a**) HRCT on the left and formalin fixed explant images of IPF lung with an apicobasal fibrotic gradient on the right. The top and bottom figures represent apical and basal locations respectively, corresponding to (**b**) the schematic picture. (**c, d**) Apical and basal lung telomere length comparison of (**c**) 8 lungs with an apicobasal gradient and (**d**) 7 lungs without apicobasal gradient measured by MMqPCR. Samples belonging to the same person are connected. Wilcoxon matched-pairs signed rank tests showed no differences in telomere length between lung sections.

**Fig 4:**
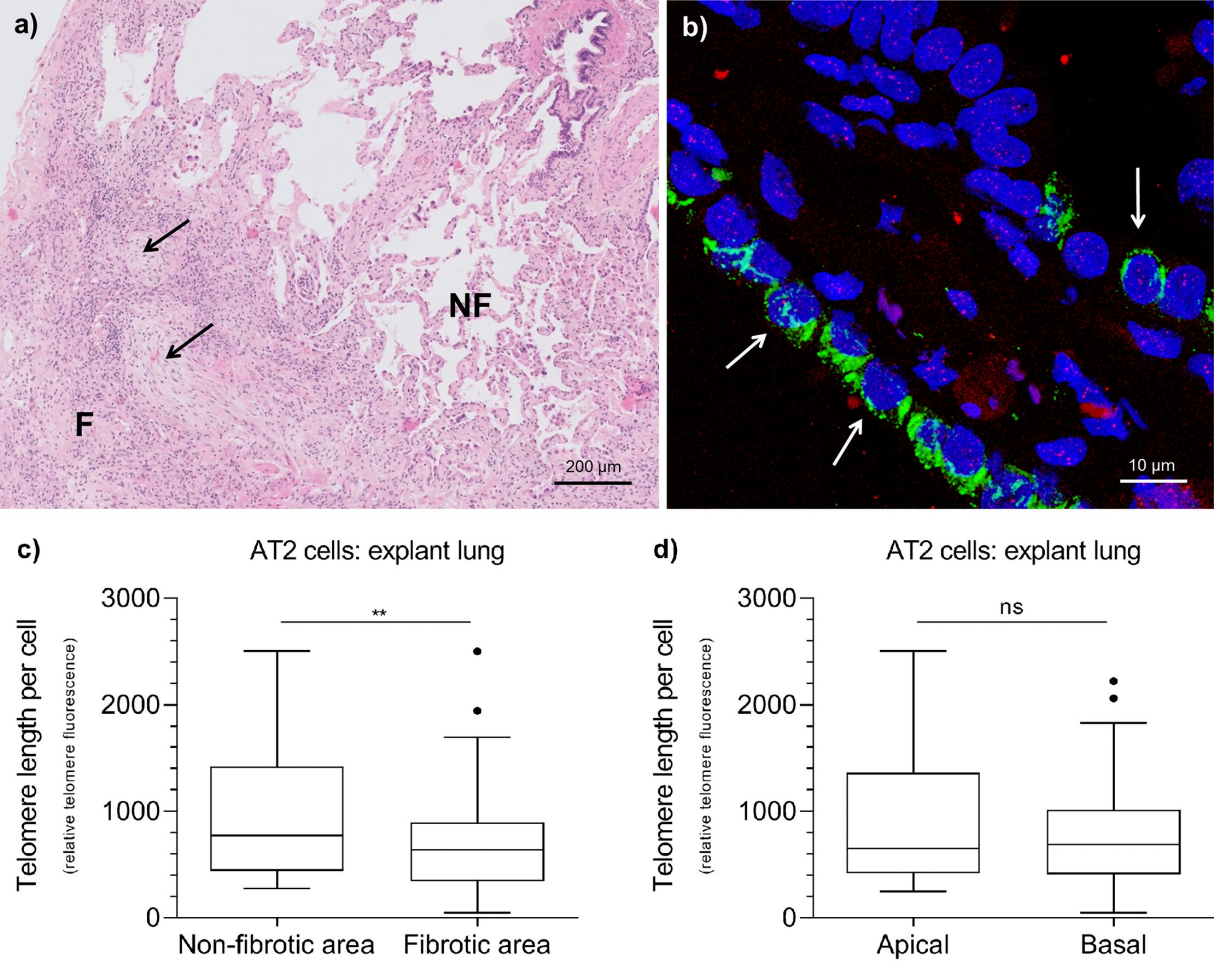
AT2 cell telomere length in IPF explant lung tissue measured by FISH. (**a**) Hematoxylin and Eosin (H&E) staining representing non-fibrotic (NF) and fibrotic (F) areas of a typical IPF lung biopsy. Black arrows indicate fibroblast foci. (**b**) Example of a combined fluorescent image of AT2 cells (white arrows) in a fibrotic area of IPF lung tissue. DNA in nucleus is displayed in blue (DAPI), proSP-C in green and telomeres in red (dots). (**c**) Within a tissue specimen, telomere length in AT2 cells was significantly longer in non-fibrotic areas than in fibrotic areas. (n = 3; Mann-Whitney test: ** < 0.01). (**d**) No difference in AT2 cell telomere length was observed between apical and basal lung tissue. Boxes represent data between 1st and 3rd quartile and whiskers extend to the highest and lowest values of expression that are not considered outliers.

Average lung telomere length was measured in apical and basal specimens from IPF explant lung with (n = 8) and without (n = 7) an apicobasal gradient on HRCT (Fig 3A and 3B). The percentage of fibrosis, measured on a macroscopic tissue level, in lungs with an apicobasal gradient was 51% in apical and 96% in basal tissue. In lungs without an apicobasal gradient these numbers were 78% for apical and 88% for basal tissue. Using MMqPCR, no significant difference in telomere length was present between basal and apical tissue (Fig 3C and 3D) and no significant differences were found between lungs with and without an apicobasal gradient (p > 0.75;). Similarly, using FISH for measurement of cell specific AT2 cell telomere length, no difference in AT2 cell telomere length between apical and basal specimens was found (p = 0.193; Fig 4D and S3 Fig).

### AT2 telomere length is short in fibrotic lesions in IPF explant lungs

Previously we showed that AT2 cell telomere length associated with fibrotic lesions in sporadic IPF diagnostic lung biopsies [25]. To verify whether this is also true for explant lungs, we performed a FISH analysis (Fig 4B) of juxtaposed microscopic non-fibrotic and fibrotic areas within three tissue specimens (Fig 4A). In non-fibrotic areas, AT2 telomere length was significantly longer than in fibrotic areas of the same biopsy (p = 0.009; Fig 4C and S3 Fig).

### A subgroup of IPF has extremely short lung telomeres

**Fig 5:**
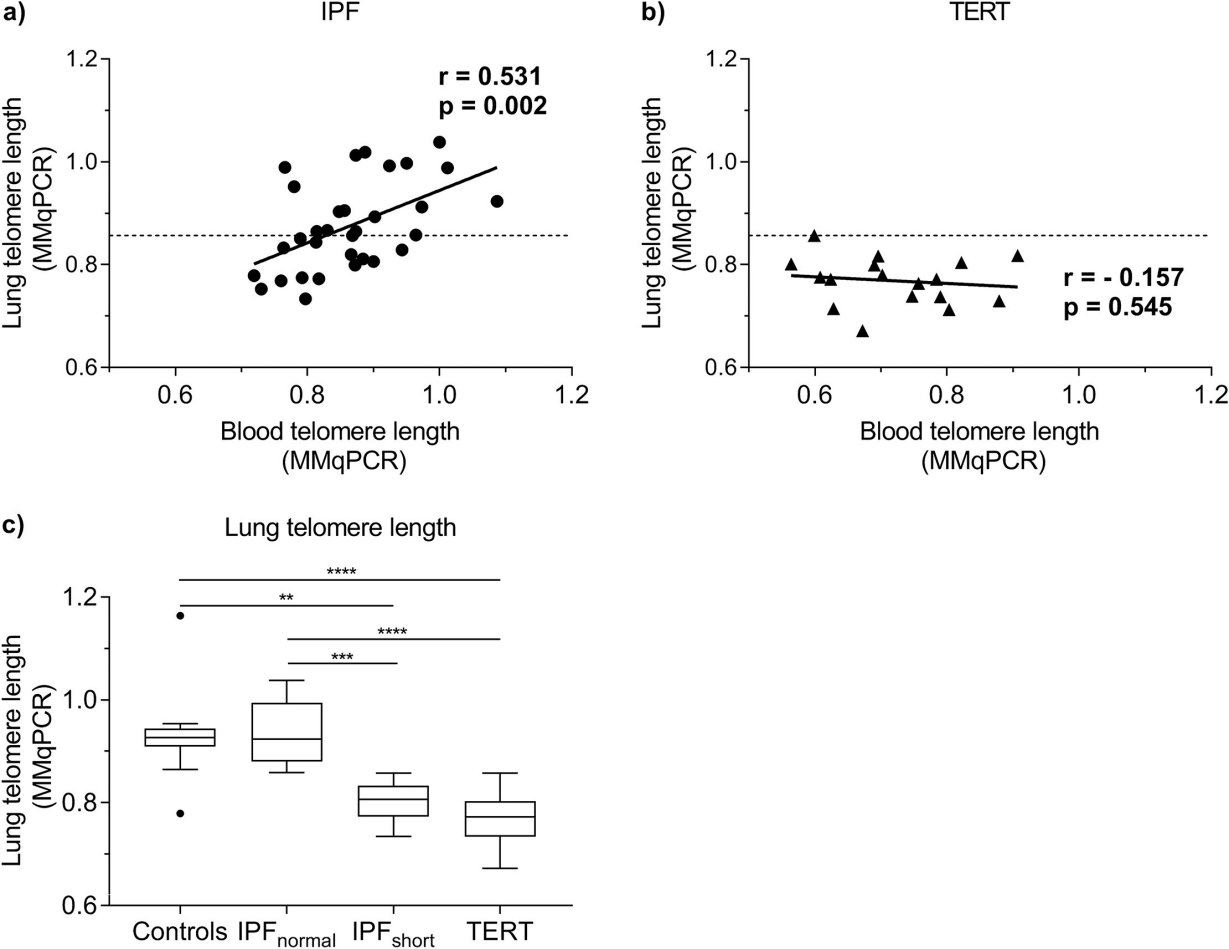
Blood and biopsy telomere length in IPF and TERT subjects measured by MMqPCR. (**a, b**) Spearman correlation of blood and biopsy telomere length in (**a**) 32 IPF and (**b**) 17 TERT cases. A significant positive correlation was established in IPF cases (r = 0.531, p = 0.002). In TERT cases, no significant correlation was found (r = -0.157, p = 0.545). Dashed lines represents threshold for lung telomere length associated with telomere related pathology. IPF_normal_ = lung telomere length above threshold, IPF_short_ = lung telomere length below threshold. (**c**) Tukey boxplots of lung telomere length measured by MMqPCR in control (n = 13), IPF_normal_ (n = 17), IPF_short_ (n = 15) and TERT (n = 17) lungs. Lung telomere length in the IPF_short_ group was significantly shorter than telomere length in age-matched controls (p = 0.0014) and IPF_normal_ (p = 0.0001). Telomere length in the TERT group was also significantly shorter than telomere length in controls (p < 0.0001) and in IPF_normal_ (p < 0.0001). Telomere length in the IPF_normal_ group and controls were comparable. Asterisks indicate significant differences calculated by Kruskal-Wallis multiple comparison tests (** = p < 0.01, *** = p < 0.001, **** = p < 0.0001). Boxes represent data between 1st and 3rd quartile and whiskers extend to the highest and lowest values of expression that are not considered outliers.

To identify a subgroup of IPF cases with possible telomere related pathology we determined blood and lung telomere length using MMqPCR in 32 sporadic IPF and 17 pulmonary fibrosis subjects with a *TERT* mutation. In IPF, blood and biopsy telomere length significantly correlated (MMqPCR, r = 0.531, p = 0.002; Fig 5A), while in TERT cases no such correlation was found (r = -0.157, p = 0.545; Fig 5B). The highest biopsy value in TERT patients served as a threshold to form an IPF_short_ group with telomere length < 0.857 (n = 15) and an IPF_normal_ group with telomere length > 0.857 (n = 17; Fig 5C). Median biopsy telomere length in IPF_short_ was, as expected, in the range of the TERT group (p > 0.999; Fig 5C). Surprisingly, median biopsy telomere length in IPF_normal_ was in the same range as that of age-matched controls. No significant differences in clinical status were observed between the patient groups (Table 1).

### AT2 cell telomere length in IPF_short_ equals that of TERT samples

**Fig 6:**
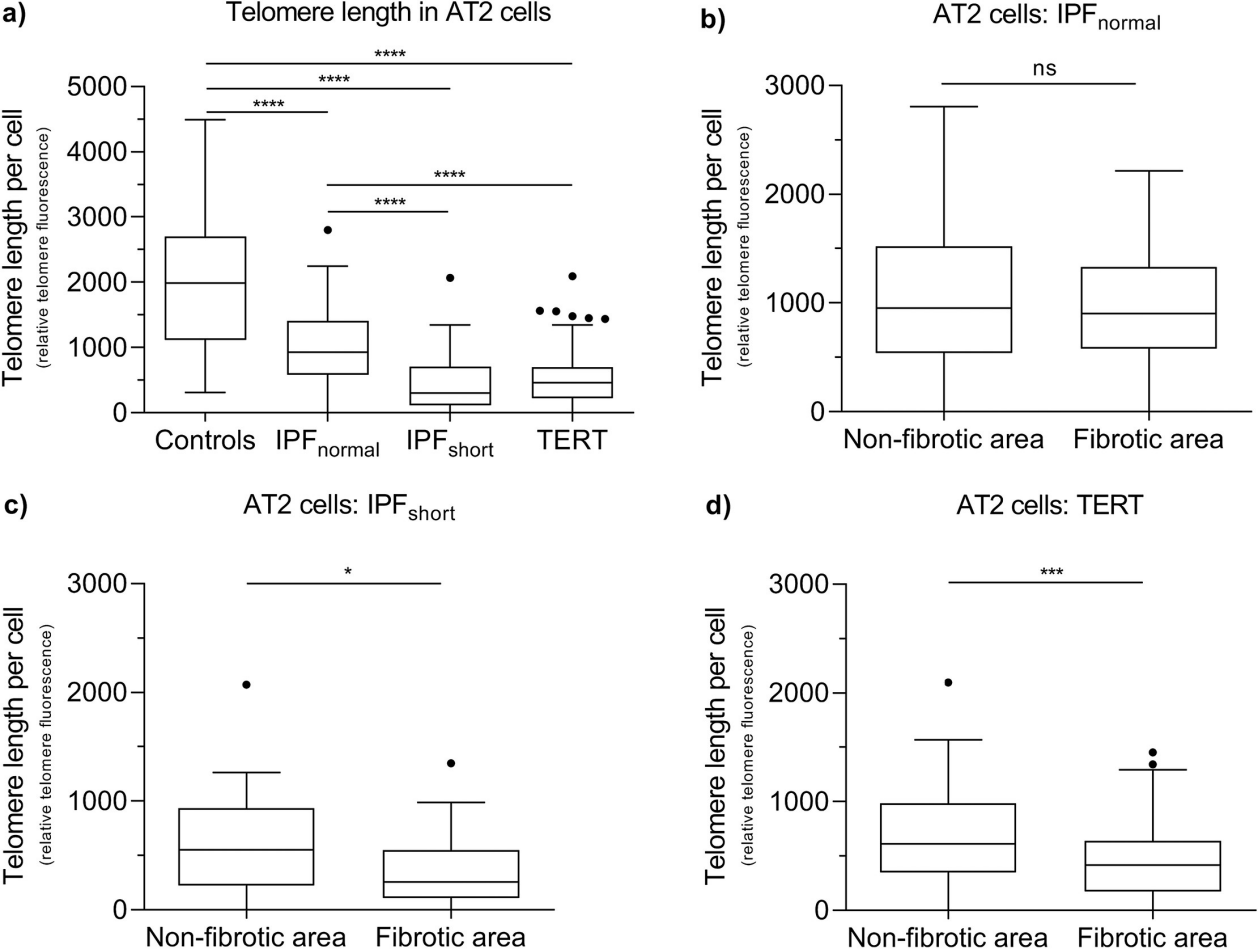
AT2 cell telomere length measured by fluorescence *in situ* hybridization (FISH) in 5 control, 5 IPF_normal_, 5 IPF_short_ and 6 TERT lungs. (**a**) Tukey box-plots of telomere length measured in AT2 cells by fluorescence in situ hybridization (FISH). All patient groups showed significant shorter AT2 cell telomere length than in controls (p < 0.0001).Telomere length in the IPF_short_ group was significantly shorter than in controls (p < 0.0001) and in IPF_normal_ (p < 0.0001). Telomere length in TERT was also significantly shorter than in controls (p < 0.0001) and in IPF_normal_ (p < 0.0001). No difference in AT2 telomere length was present between IPF_short_ and TERT. Asterisks indicate significant differences calculated by Kruskal-Wallis multiple comparison tests. (**b, c, d**) AT2 cell telomere length in non-fibrotic and fibrotic lung areas in (**b**) IPF_normal_, (**c**) IPF_short_ and (**d**) TERT lungs. In IPF_normal_ no difference was found between areas. AT2 cell telomere length in non-fibrotic areas was significantly longer than fibrotic areas in IPF_short_ (p = 0.0237) and TERT (p = 0.0001) lungs. Asterisks indicate significant differences calculated by Mann-Whitney analyses (ns = not significant, * = p < 0.05, *** = p < 0.001, **** = p < 0.0001). Boxes represent data between 1st and 3rd quartile and whiskers extend to the highest and lowest values of expression that are not considered outliers.

Next, we determined the distribution of telomere length of AT2 cells in the IPF_normal_, IPF_short_ and TERT groups with FISH staining. AT2 cell telomere length in IPF_normal_ and IPF_short_ patient groups were respectively 2.1 and 6.5 times shorter than in controls (p < 0.0001; Fig 6A and S3 Fig). Comparison between the patient groups showed that AT2 cell telomere length in IPF_normal_ was 2 times longer than in the TERT group, while no difference between the IPF_short_ and the TERT group was found.

In a more detailed analysis, comparison of AT2 cell telomere length in fibrotic versus non-fibrotic areas of the same biopsy (Fig 4A) showed that only in IPF_short_ and TERT biopsies the telomere length of AT2 cells in fibrotic areas was shorter than in non-fibrotic areas (p = 0.024 and p < 0.001 respectively; Fig 6C and 6D). In contrast, in IPF_normal_ no significant difference was observed (p = 0.722; Fig 6B and S3 Fig).

### Whole exome sequencing in IPF

To identify mutations in telomere related genes associated with pulmonary fibrosis, we decided to perform whole exome sequencing in all 32 sporadic IPF patients. Sequencing data revealed 3 telomere-related variants, all in the IPF_short_ group, including a stop gain (c.3028C>T) and a missense (c.2258G>A) variant in *RTEL1* and a missense (c.1310G>A) variant in *PARN* (Table 2). High CADD scores for both *RTEL1* variants indicated that these were pathogenic, while the CADD score of 22 for the *PARN* mutation implied a trend towards pathogenicity as reported by the GAVIN method.

**Table 2:** Telomere-related genetic variants discovered in IPF cases by WES.

*Lung telomere length (MMqPCR)*	0.857 (IPF_short_)	0.798 (IPF_short_)	0.829 (IPF_short_)
*Gene*	*RTEL1*	*RTEL1*	*PARN*
*Chromosome*	20	20	16
*Position GRCh37/hg19*	g.62324600	g.62321484	g.14649519
*Annotation*	c.3028C>T (NM_032957.4) p.(Arg1010Ter) (NP_116575.3)	c.2258G>A (NM_032957.4) p.(Arg753His) (NP_116575.3)	c.1310G>A (NM_002582.4) p.(Gly437Glu) (NP_002573)
*Reference*	rs373740199	rs777153220	N/A
*Effect*	Stop gain	Missense	Missense
*ACMG classification* [30]	Likely pathogenic	Uncertain significance	Uncertain significance
*CADD score (minimum value for pathogenicity)*	35 (19.35)	27.2 (19.35)	33 (34)
*SIFT prediction*	N/A	Damaging	Damaging
*PolyPhen-2 prediction*	N/A	Probably damaging	Probably damaging
*ExAC %*	0.008	0.001	0
*Literature*	[31,32]		

IPF = Idiopathic Pulmonary Fibrosis; CADD = Combined Annotation Dependent Depletion score; ExAC = Exome Aggregation Consortium

PolyPhen-2 = Polymorphism Phenotyping version 2; SIFT = Sorting Intolerant From Tolerant; N/A = Not Applicable.

Refsnp (rs) single-nucleotide polymorphism identifiers are provided where available. Pathogenicity of CADD scores were interpreted using the Gene-Aware Variant INterpretation (GAVIN) method (minimum value needed for pathogenicity is noted between brackets).

## Discussion

Aberrant maintenance of telomere length is accepted as a possible cause of pulmonary fibrosis in familial and sporadic IPF. Patients with short telomeres showed short median survival of 22 months, which was even shorter than that generally found in IPF [25]. Nonetheless, little is known about organ telomere length in IPF. Previous studies showed that telomeres were relatively short in skin, thyroid and lung tissues of healthy macaques [24]. In human controls, skin and liver samples had relatively short telomeres, while lung and thyroid were not evaluated [20]. In this study, we show that the lung of three IPF subjects had the shortest telomere length among 5 organs samples, while this was not found in the two control lungs. This may be indicative of accelerated aging of fibrotic IPF lungs and the reason why IPF patients only suffer from a diseased lung. The finding provides rationale for further investigation of the association of lung telomere length and fibrosis in IPF lung.

Telomere shortening is a hallmark of aging and just recently it was shown that, similar to other organs, telomeres in control lungs shorten with age [26]. However, lung telomere length in our IPF samples was shorter than in age-matched controls and did not correlate with age. Similar findings are known from patients suffering from bone-marrow failure due to telomere related gene mutations in which leukocyte telomere length no longer correlates with age [33]. In addition, we found that liver telomeres were short in both IPF and controls. Even though liver cirrhosis is also described to be a consequence of telomere-related defects [34,35], no signs of chronic liver disease were found in our group.

Next, by comparing diagnostic biopsy samples with samples from explant lungs, we were able to follow the evolution of telomere length during the fibrosis. To our surprise, no difference in average telomere length was observed over the course of the disease. This implies that genetic or environmental factors must have added significantly to telomere shortening prior to disease diagnosis. In addition, it is also still possible that long-term asymptomatic disease is responsible for the observed shortening. At diagnosis, IPF patients already have severely impaired diffusing capacity for carbon monoxide (DLCO), averaging around 50% of predicted [36] and it is unknown at what point in time the causative pathogenic processes initiated.

In paired samples from control lung it was recently demonstrated that telomeres in basal regions are shorter than in apical regions of the lung [26]. IPF lung characteristically present a UIP pattern on HRCT with an apicobasal fibrotic gradient. When we assessed telomere length in IPF explant lungs with and without this gradient, no difference between apical and basal lung telomere length was found, irrespective of presence of the apicobasal gradient. Thus in IPF, the effects of natural aging on lung telomere length are completely masked. Because differences in average lung telomere length in time and location are absent in IPF, it is likely that the duration of the presence of short telomeres associates with the degree of fibrosis. Previously it was reported that short telomeres induce cellular senescence, thereby signalling the pro-fibrotic senescence-associated secretory phenotype (SASP) [8–10]. Therefore, as cellular senescence is not resolved, fibrotic remodelling may accumulate over time.

The current concept of IPF pathogenesis describes that AT2 cells, alveolar progenitor cells with high division capacity, play a fundamental role in the onset of fibrogenesis [37,38]. This is supported by studies in which mice with selectively knocked out telomere repeat binding factor-1 (TRF-1) in AT2 cells were prone to develop pulmonary fibrosis [39].

In our IPF cohort, average lung telomere length proved to be highly variable. Grouping the sporadic IPF patients with short–TERT-like–lung telomere length together, showed that they had very short AT2 cell telomere length, particularly in fibrotic lesions. Because the TERT group also demonstrated AT2 cell telomere shortening predominantly in fibrotic lesions, this so-called IPF_short_ subgroup might be underlying telomere driven disease. A previous study investigating the cell proliferation marker Ki-67 showed that elevated cell proliferation is likely not the cause of telomere shortening in IPF [40]. Furthermore, 3 patients with telomere related gene mutations were found in this group, supporting the likelihood of telomere related disease pathogenesis. The *RTEL1* c.3028C>T sequence variant was previously associated with IPF, dyskeratosis congenita and Hoyeraal-Hreidarsson Syndrome [31,32]. Recently an *RTEL1* c.2257C>T variant resulting in an amino acid substitution at the same codon as our c.2258G>A variant was described in a patient with pulmonary fibrosis [32]. Moreover, *in silico* prediction models such as high CADD scores, SIFT and Polyphen-2 predictions and low ExAc frequencies support pathogenicity. The relatively high number of variants in *PARN* and *RTEL1* in our cohort was justified by previous data in which 11.3% of a sporadic IPF cohort harboured qualifying genetic variant in *TERT*, *RTEL1* or *PARN* [32].

Most interestingly, the remaining group with lung telomere length above the range of TERT cases, had normal average lung telomere length comparable with those in controls. And although AT2 telomere length in this so-called IPF_normal_ group was shorter than in controls, it was significantly longer than in the TERT and IPF_short_ group. This is highly suggestive of a disease pathogenesis other than telomere driven disease, which may take a toll on AT2 proliferative capacity and is driven by other pro-fibrotic processes. We found a higher number of pack years in the IPF_short_ group, which would be suggestive of environmental driven disease, however, the difference was not significant (p = 0.364). Therefore, other processes involving alveolar homeostasis may be involved.

Some limitations of the study are worth noting. The data presented here are based on associations; no causative links could be concluded from telomere length or WES analysis. Next, control tissue was obtained from various sources, such as residual lung resected from tissue next to a tumour. Also, MMqPCR was used to assess telomere length in the lung. Although telomere restriction fragment (TRF) length analysis is often used for tissue analysis, literature showed that a strong correlation exists between TRF, MMqPCR and FISH [29]. Furthermore, we included a relatively young IPF case of 35 years old. However, since the WES analysis for IPF-related genes was negative and no family history of disease was known, the subject was classified as sporadic IPF.

In conclusion, this study demonstrates that there is no difference in average lung telomere length in time and location, which make it plausible that lung telomere shortening occurs prior to disease diagnosis. This opens up possibilities for early detection of patients at risk of developing IPF if easily accessible markers would be available that associate with this process. Furthermore, a subgroup of patients with IPF shows similarities with subjects harbouring a *TERT* mutation. 20% of this IPF group have telomere related gene mutations. These patients have extremely short AT2 telomeres in fibrotic lesions and may have telomere driven pathology. On the other hand, IPF patients with lung telomere length above the range of TERT patients have normal lung telomere length, and mildly short AT2 cells. In these patients disease is unlikely to be telomere driven but might associate with other processes that affect AT2 cell turnover. Future measurement of lung telomere length in IPF may aid discrimination between telomere-related and telomere-unrelated pulmonary fibrosis.

## Supporting information

S1 Fig Schematic overview of the three subject groups included in this study. In total 82 individual cases were included. No overlapping cases were present between the groups. Patients were distributed over three groups based on origin of the tissue (autopsies in group 1, diagnostic biopsies in group 2 and explant lung in group 3). MMqPCR = Monochrome multiplex quantitative polymerase chain reaction; FISH = Fluorescence *in situ* hybridization; HRCT = High-resolution computed tomography.
(TIF)

S2 Fig Telomere length in control lung. A trend towards a significant Spearman correlation was found between lung telomere shortening measured by MMqPCR and increasing age (n = 18, r = -0.4, p = 0.115).
(TIF)

S3 Fig Raw data of the AT2 cell telomere length in explant lungs and diagnostic biopsies. (**a, b**) Telomere length of AT2 cells in explant lungs measured by FISH. (**a**) Within explant lung specimens, telomere length in AT2 cells was significantly longer in non-fibrotic areas than in fibrotic areas. (n = 3; Mann-Whitney test: ** < 0.01). (**b**) No difference in AT2 cell telomere length was observed between apical and basal lung tissue. (**c, d, e, f)** Telomere length of AT2 cells in diagnostic biopsies measured by FISH. (**c**) All patient groups showed significant shorter AT2 cell telomere length than in controls (p < 0.0001).Telomere length of IPF_short_ was significantly shorter than in controls (p < 0.0001) and in IPF_normal_ (p < 0.0001). Telomere length in TERT was also significantly shorter than in controls (p < 0.0001) and in IPF_normal_ (p < 0.0001). No difference in AT2 telomere length was present between IPF_short_ and TERT. Asterisks indicate significant differences calculated by Kruskal-Wallis multiple comparison tests. (**b, c, d**) AT2 cell telomere length in non-fibrotic and fibrotic lung areas in (**b**) IPF_normal_, (**c**) IPF_short_ and (**d**) TERT lungs. In IPF_normal_ no difference was found between areas. AT2 cell telomere length in non-fibrotic areas was significantly longer than fibrotic areas in IPF_short_ (p = 0.0237) and TERT (p = 0.0001) lungs. Asterisks indicate significant differences calculated by Mann-Whitney analyses (ns = not significant, * = p < 0.05, *** = p < 0.001, **** = p < 0.0001). Every dot represents an individual AT2 cell and each subject is indicated by a different colour.
(TIF)

S4 Fig Comparison of MMqPCR and AT2 cell FISH results. Telomere length measurements in lungs of 10 IPF, 6 TERT and 5 control subjects by MMqPCR and FISH. A significant spearman correlation was found between both techniques for AT2 cell telomere length in fibrotic (red symbols; r = 0.808, p < 0.001) and in non-fibrotic areas (black symbols: r = 0.612, p = 0.003). Note that controls do not contain fibrotic areas.
(TIF)

S1 File Supplemental methods and results. (DOCX)

S1 Table Characteristics of controls, explant lungs and autopsies. (DOCX)
