# Supplementary figures and images for: From organ to cell: Multi-level telomere length assessment in patients with idiopathic pulmonary fibrosis
Source: PLoS One. 2020 Jan 7;15(1):e0226785. doi: 10.1371/journal.pone.0226785 (PMC6946122; doi:10.1371/journal.pone.0226785)

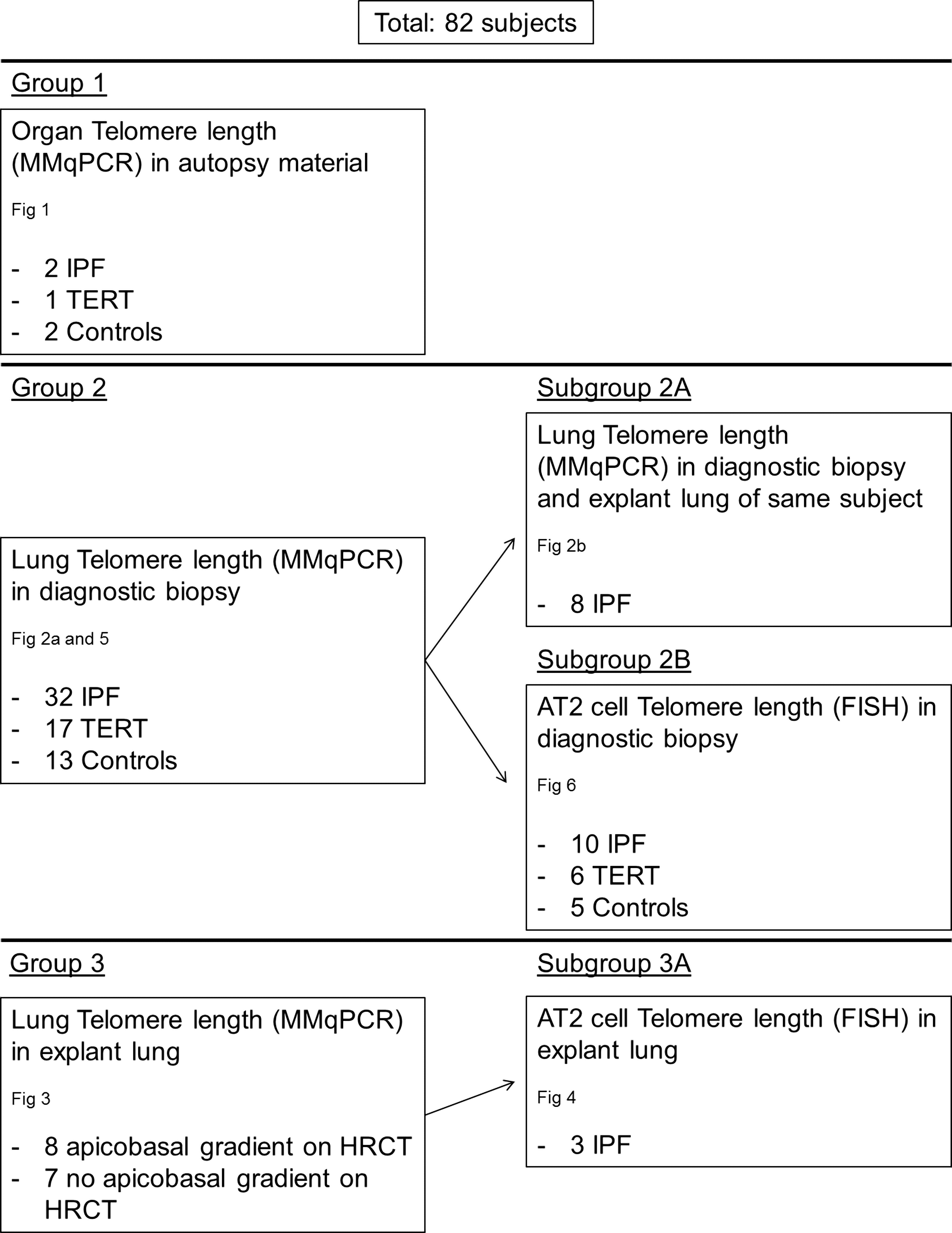

Supplement: S1 Fig — In total 82 individual cases were included. No overlapping cases were present between the groups. Patients were distributed over three groups based on origin of the tissue (autopsies in group 1, diagnostic biopsies in group 2 and explant lung in group 3). MMqPCR = Monochrome multiplex quantitative polymerase chain reaction; FISH = Fluorescence in situ hybridization; HRCT = High-resolution computed tomography. (TIF) [file pone.0226785.s001.tif]

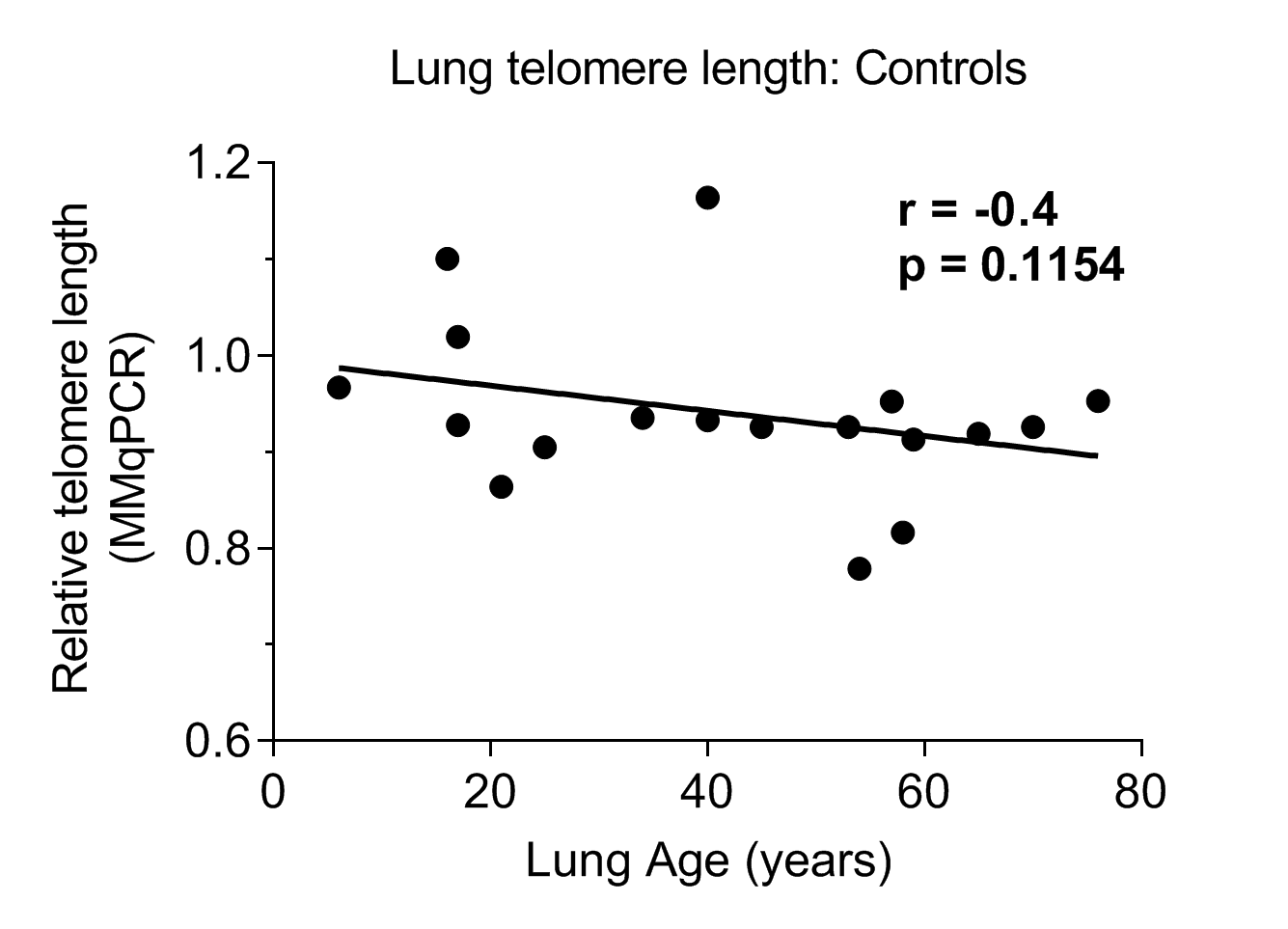

Supplement: S2 Fig — A trend towards a significant Spearman correlation was found between lung telomere shortening measured by MMqPCR and increasing age (n = 18, r = -0.4, p = 0.115). (TIF) [file pone.0226785.s002.tif]

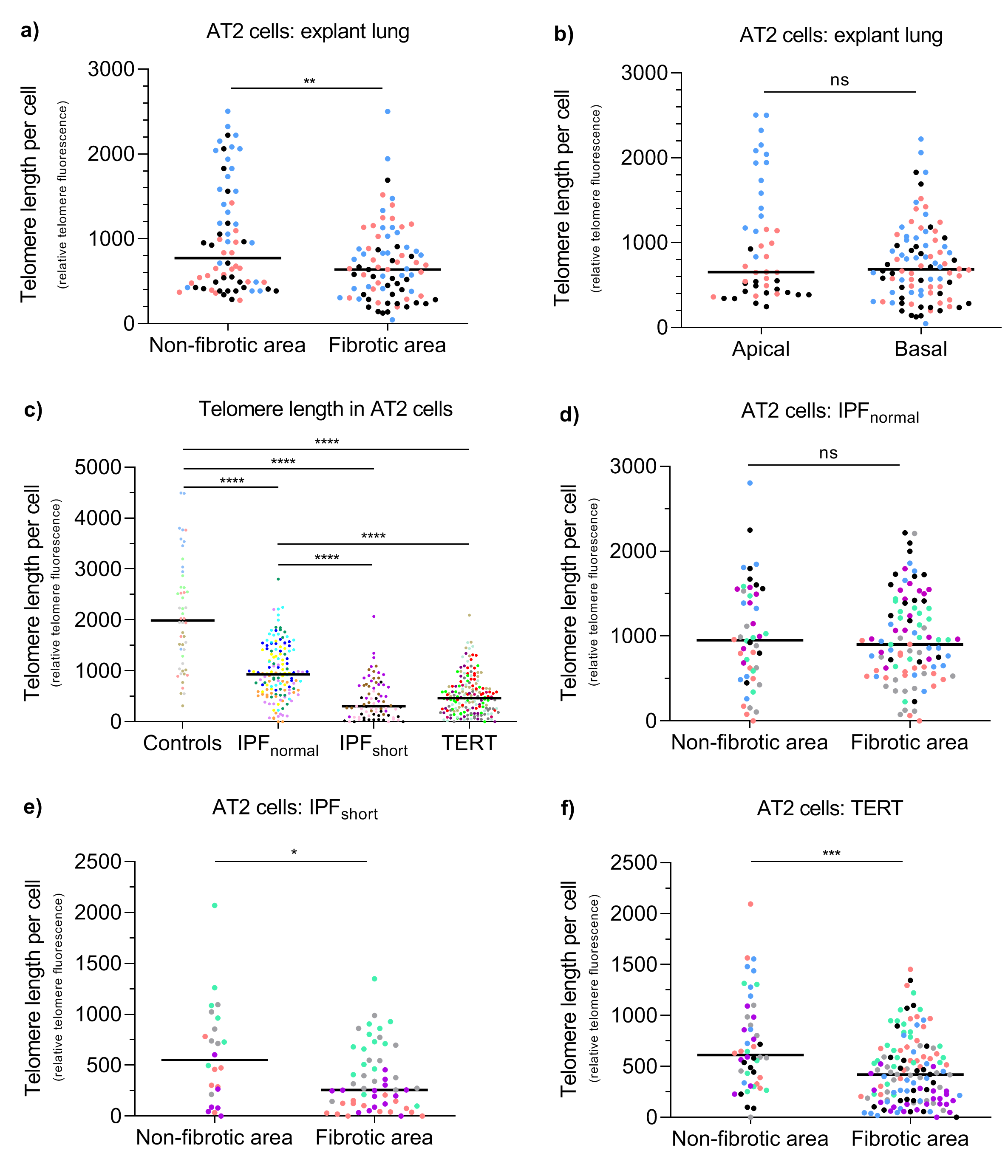

Supplement: S3 Fig — (a, b) Telomere length of AT2 cells in explant lungs measured by FISH. (a) Within explant lung specimens, telomere length in AT2 cells was significantly longer in non-fibrotic areas than in fibrotic areas. (n = 3; Mann-Whitney test: ** < 0.01). (b) No difference in AT2 cell telomere length was observed between apical and basal lung tissue. (c, d, e, f) Telomere length of AT2 cells in diagnostic biopsies measured by FISH. (c) All patient groups showed significant shorter AT2 cell telomere length than in controls (p < 0.0001).Telomere length of IPFshort was significantly shorter than in controls (p < 0.0001) and in IPFnormal (p < 0.0001). Telomere length in TERT was also significantly shorter than in controls (p < 0.0001) and in IPFnormal (p < 0.0001). No difference in AT2 telomere length was present between IPFshort and TERT. Asterisks indicate significant differences calculated by Kruskal-Wallis multiple comparison tests. (b, c, d) AT2 cell telomere length in non-fibrotic and fibrotic lung areas in (b) IPFnormal, (c) IPFshort and (d) TERT lungs. In IPFnormal no difference was found between areas. AT2 cell telomere length in non-fibrotic areas was significantly longer than fibrotic areas in IPFshort (p = 0.0237) and TERT (p = 0.0001) lungs. Asterisks indicate significant differences calculated by Mann-Whitney analyses (ns = not significant, * = p < 0.05, *** = p < 0.001, **** = p < 0.0001). Every dot represents an individual AT2 cell and each subject is indicated by a different colour. (TIF) [file pone.0226785.s003.tif]

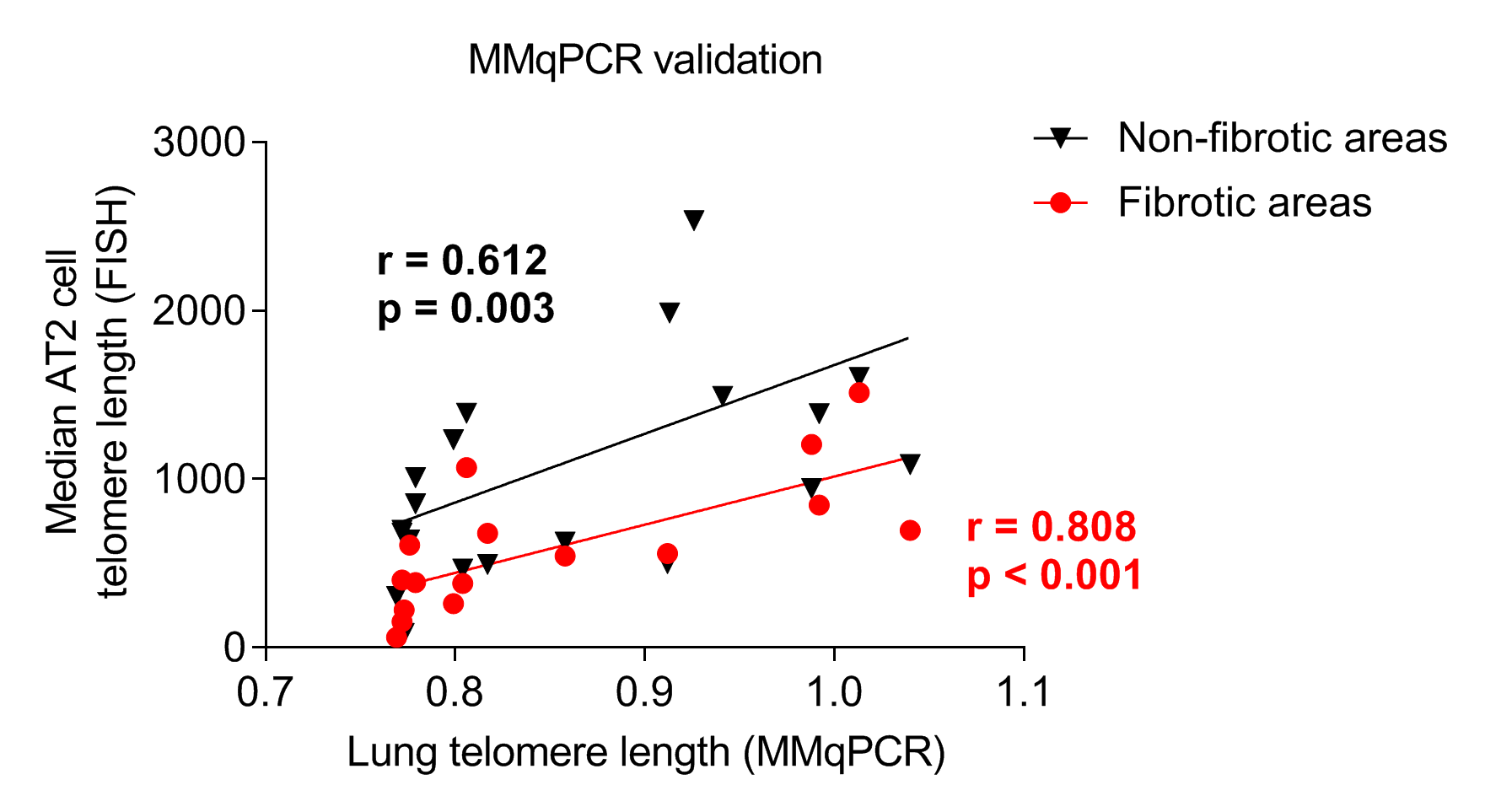

Supplement: S4 Fig — Telomere length measurements in lungs of 10 IPF, 6 TERT and 5 control subjects by MMqPCR and FISH. A significant spearman correlation was found between both techniques for AT2 cell telomere length in fibrotic (red symbols; r = 0.808, p < 0.001) and in non-fibrotic areas (black symbols: r = 0.612, p = 0.003). Note that controls do not contain fibrotic areas. (TIF) [file pone.0226785.s004.tif]
