# Supplementary material for: From organ to cell: Multi-level telomere length assessment in patients with idiopathic pulmonary fibrosis
Source: PLoS One. 2020 Jan 7;15(1):e0226785. doi: 10.1371/journal.pone.0226785 (PMC6946122; doi:10.1371/journal.pone.0226785)
Supplement: S1 File — (DOCX) [file pone.0226785.s005.docx]

**S1 File. Supplemental methods and results.**

**From Organ to Cell: Multi-level Telomere Length Assessment in Patients with Idiopathic Pulmonary Fibrosis**

Aernoud A. van Batenburg, Karin M. Kazemier, Matthijs F.M. van Oosterhout, Joanne J. van der Vis, Wouter H. van Es, Jan C. Grutters, Roel Goldschmeding and Coline H.M. van Moorsel

**Supplemental methods**

**Patient selection and clinical data collection**

Patient inclusion for this study is presented in S1 Fig. Subject characteristics were included in Table 1 and S1 Table. 20 cases were included in our previous publication [1]. However, in the current study all FISH and MMqPCR experiments were original for every subject. In the TERT group, 15 out of 17 cases were found in the context of familial pulmonary fibrosis. Familial disease was determined when 2 or more first-degree family members also presented with pulmonary fibrosis. The remaining two *TERT* patients were discovered because of the relatively young age at which they were diagnosed with IPF. Patient characteristics were retrieved from medical reports between 6 months before and after date of biopsy. Moreover, in order to measure telomere length in apical and basal areas of the lung 8 IPF explant lungs with an apicobasal gradient and 7 explant lungs without an apicobasal gradient on High-resolution computed tomography (HRCT). HRCT scans within 6 months before transplantation were included. The mean fibrosis rate in these apical and basal specimens was measured by the amount of white fibrotic tissue as a percentage of the total tissue. As controls, lung tissue with no pathology, obtained during post-mortem examination (n = 8), from residual donor lobes (n = 3) and relatively normal lung resected from tissue next to a tumour (n = 7) were used. The age ranged from 6 to 76 years old. For comparison of control lung telomere length with that of subjects with pulmonary fibrosis, controls were age matched (n = 13). To assess telomere length of multiple organs of control and IPF patients, residual lung, kidney, thyroid, liver and bladder tissues were included from autopsy section material. One of the three subjects (IPF 1 in Fig 1) in this study died because of an acute exacerbation. Control autopsies were age-matched to subjects with pulmonary fibrosis and did not have lung related pathology, but died of heart failure (n = 2).

**Telomere length measurements by MMqPCR in FFPE tissue and peripheral blood leukocytes**

Genomic DNA was extracted from peripheral leukocytes using a magnetic beads-based method (chemagic DNA blood 10k kit; Perkin Elmer Inc. Waltham, MA, USA). In the case of FFPE tissue, DNA was isolated using an AllPrep DNA/RNA FFPE Kit (Qiagen, Hilden, Germany) according to manufacturer instructions. Slides were cut from sequential sections used for FISH. The paraffin was removed using paraffin dissolver (Macherey-Nagel, Düren, Germany). DNA was quantified using a Nanodrop (Thermo Fisher Scientific, Waltham, MA, USA) and sample purity was determined using the absorbance ratio of 260 and 280nm. Samples within a ratio of 1.8 - 2.0 were included. To measure whole lung biopsy and leukocyte telomere length, monochrome multiplex qPCR (MMqPCR) was performed as described previously [4,5]. Because amplification of telomere and β-globin in FFPE DNA is delayed compared to blood derived DNA we adjusted cycle counts for all FFPE samples with -5 and -7 respectively. The relative telomere length for each sample was estimated from the ratio telomere repeat copy number (T) to a single human β-globin gene copy number (S) (T/S ratio), using standard curves from a serial dilution of a genomic DNA-pool [4]. Quadruplicate reactions were performed on a MyiQ™ Single-Color Real-Time PCRDetection System (Bio-Rad, Hercules, CA, USA) using iQ SYBR Green Supermix (Bio-Rad, Hercules, CA, USA). Samples were analysed in triplicate in at least two runs, of which only coefficients of variation below 10%, with an overall mean of 2.5, were included.

**AT2 cell telomere length measurements with fluorescence *in situ* hybridization (FISH)**

In short, FFPE slides were pre-treated with a trisaminomethane-ethylenediaminetetraacetic acid solution (Tris-EDTA; 40-mM Tris, 1-mM EDTA, pH 9) for 20 min. Slides were incubated overnight at 37 ^0^C with a telomere-Cy3 peptide nucleotide acid (PNA) probe (2.70 μg/ml, F1002; Panagene, Daejeon, South Korea) which was diluted in a 2x saline-sodium citrate buffer (SSC) (0.3-M NaCl, 0.03-M sodium citrate), 5% dextran sulphate, 50% deionized formamide, and 0.5% Tween-20 hybridization mixture. Excess probe was cleared with a PNA wash solution (1% 1-M Tris, 29% Aquadest, and 70% Formamide). Next, to identify AT2 cells, a rabbit anti-human proSP-C antibody (AB3786; Merck Millipore) was applied 1:100 in 1% BSA/PBS and incubated 1 hr at room temperature. Slides were incubated with a secondary Goat anti-rabbit Alexa Fluor 488 (1:80 in 1% BSA/PBS, A11008, Thermo Fisher Scientific) antibody for 30 min at room temperature. Subsequently,

4′,6-diamidino-2-phenylindole (DAPI; 25 μg/ml) was used to stain nuclei. Pictures were taken with a LSM700 laser scanning confocal microscope (Zeiss, Jena, Germany) and images were analysed using the image analysis Telometer plugin (available at http://demarzolab.pathology.jhmi.edu/telometer/index.html) of ImageJ (<http://rsb.info.nih.gov/ij/>).

**Whole exome sequencing bioinformatics pipeline**

Whole exome sequence data was analysed with Ingenuity Variant Analysis (version 5.4.20181019, Qiagen, Hilden, Germany) using the following filters: variants were required to 1) have a call and genotype quality of at least 30, 2) have a read depth of at least 15, 3) be outside top 5% most exonically variable 100base windows in healthy public genomes, 4) be excluded when an allele frequency of at least 1% was observed in the 1000 genomes project, ExAC, gnomAD and NHLBI ESP exomes populations unless an established pathogenic common variant was known and 5) have no more than 20 bases in an intron that i) are experimentally observed to be associated with a pathogenic, likely pathogenic or an uncertain significance phenotype according to computed ACMG Guidelines classification [6] (or are listed in HGMD®) or ii) are associated with gain of function of a gene established in the literature or is inferred activating mutation by Ingenuity or iii) are associated with loss of function by frameshift, start/stop codon change, missense, splice site loss up to 2 bases into intron (or as predicted by MaxEntScan), copy number or promotor loss. Finally, variants were only selected when located within the telomere-related genes *TERT, RTEL1, TINF2, PARN, DKC1, TERC* or *NAF1*. Combined Annotation Dependent Depletion (CADD) scores were interpret using the Gene-Aware Variant INterpretation (GAVIN) method [7].

**Supplemental results**

**Telomere length measured by MMqPCR validation by FISH**

We compared MMqPCR with AT2 cell FISH in the same FFPE biopsies of 10 IPF, 6 TERT and 5 control cases. Spearman correlation showed a good correlation between the two methods for fibrotic (r = 0.808, p < 0.001, S4 Fig) and non-fibrotic areas (r = 0.612, p = 0.003, S4 Fig).

**References**

[1]Snetselaar R, van Batenburg AA, van Oosterhout MFM, Kazemier KM, Roothaan SM, Peeters T, et al. Short telomere length in IPF lung associates with fibrotic lesions and predicts survival. PLoS One 2017 Dec 27;12(12):e0189467.

[2]Raghu G, Collard HR, Egan JJ, Martinez FJ, Behr J, Brown KK, et al. An official ATS/ERS/JRS/ALAT statement: idiopathic pulmonary fibrosis: evidence-based guidelines for diagnosis and management. Am J Respir Crit Care Med 2011 Mar 15;183(6):788-824.

[3]Travis WD, Costabel U, Hansell DM, King TE,Jr, Lynch DA, Nicholson AG, et al. An official American Thoracic Society/European Respiratory Society statement: Update of the international multidisciplinary classification of the idiopathic interstitial pneumonias. Am J Respir Crit Care Med 2013 Sep 15;188(6):733-748.

[4]Cawthon RM. Telomere length measurement by a novel monochrome multiplex quantitative PCR method. 2009 feb;37(3):e21.

[5]Snetselaar R, van Moorsel CH, Kazemier KM, van der Vis JJ, Zanen P, van Oosterhout MF, et al. Telomere length in interstitial lung diseases. Chest 2015 Oct;148(4):1011-1018.

[6]Richards S, Aziz N, Bale S, Bick D, Das S, Gastier-Foster J, et al. Standards and guidelines for the interpretation of sequence variants: a joint consensus recommendation of the American College of Medical Genetics and Genomics and the Association for Molecular Pathology. Genet Med 2015 May;17(5):405-424.

[7]van der Velde KJ, de Boer EN, van Diemen CC, Sikkema-Raddatz B, Abbott KM, Knopperts A, et al. GAVIN: Gene-Aware Variant INterpretation for medical sequencing. Genome Biol 2017 Jan 16;18(1):6-016-1141-7.
