# Supplementary material for: From organ to cell: Multi-level telomere length assessment in patients with idiopathic pulmonary fibrosis
Source: PLoS One. 2020 Jan 7;15(1):e0226785. doi: 10.1371/journal.pone.0226785 (PMC6946122; doi:10.1371/journal.pone.0226785)
Supplement: S1 Table — (DOCX) [file pone.0226785.s006.docx]

**S1 Table. Characteristics of controls, explant lungs and autopsies**

|  | Controls | Explant lungs | Autopsies |
| --- | --- | --- | --- |
| N | 15 | 15 | 3 |
| Male/Female | 11/4 | 8/7 | 3/0 |
| Biopsy Age (SD) | 50.3 (16.2) | 57.5 (9.3) | 69 (11.5) |
| Mean FVC%pred (SD) | N/A | 44.2 (17.9) | 78.5 (10) |
| Mean DLCO%pred (SD) | N/A | 30.1 (12.3) | 36.3 (12.4) |
| Smoke status (CS:FS:NS:U) | N/A | 0:13:2:0 | 0:1:2:0 |
| Pack years | N/A | 23 (21.2) | 5 (8.7) |

FVC = Forced Vital Capacity; DLCO = Diffusing Capacity of the Lungs for Carbon Monoxide. Pred = Predicted; CS = Current Smoker; FS = Former Smoker; NS = Never Smoker; U = Unknown; N/A = Not Applicable
